# Comparative Efficacy of Tumor Microenvironment-responsive Nanotherapeutics Targeting PSD95/Discs-large/ZO-1 Binding Kinase in Different Histological Subgroups of Medulloblastoma

**DOI:** 10.7150/ijms.97992

**Published:** 2024-11-11

**Authors:** Qi Zhang, Chao Hu, Baoqing Qu, Cuiping Zhang, Longtao He

**Affiliations:** 1 Department of Ultrastructural Pathology, Beijing Neurosurgical Institute/ Beijing Tiantan Hospital, Capital Medical University, Beijing, 100070, China.; 2 Department of Breast Surgery, Beijing Luhe Hospital, Capital Medical University, Beijing, 101100, China.

**Keywords:** tumor microenvironment-responsive nanocomposite, PDZ-binding kinase, medulloblastoma, glutathione, rabies virus glycoprotein

## Abstract

This work aimed to demonstrate the therapeutic effects of tumor microenvironment-responsive nanotherapeutic drugs targeting PSD95/Discs-large/ZO-1 domain (PDZ)-binding-kinase (PBK) in medulloblastoma Daoy and ONS-76 cells. The objective was to provide critical theoretical and practical foundations for the clinical adoption of tumor microenvironment-responsive nanotherapeutic drugs targeting PBK. The rabies virus glycoprotein (RVG) was utilized as a specific targeting molecule to form a tumor microenvironment-responsive nanocomplex, HPAA/RVG/PBK-siRNA, which incorporated glutathione (GSH) as a microenvironment stimulus factor within a hyperbranched polymer polyamide amine (HPAA). This nanocomplex also carried PBK-small interfering RNA (siRNA) for targeted PBK therapy. Characterization of HPAA, maleimide-polyethylene glycol-N-succinyl ester (MAL-PEG-NHS), HPAA-PEG, RVG, HPAA-RVG, and HPAA/RVG/PBK-siRNA was conducted using nuclear magnetic resonance spectroscopy, high-performance liquid chromatography (HPLC), dynamic light scattering, and transmission electron microscopy (TEM). Flow cytometry was employed to assess endocytosis and cell transfection of HPAA-RVG and HPAA/RVG/PBK-siRNA in Daoy and ONS-76 cells. The two cell lines were treated with HPAA/RVG/PBK-siRNA (HPAA/siRNA group), methoxy-PEG polyethylenimine (PEI-25k)/PBK-siRNA (PEI group), HPAA/RVG nanocarriers without PBK-siRNA (HPAA/RVG group), Dharmacon™ non-targeting siRNA (shNTC group), PBK-siRNA (Control group 1), AChR inhibitor (Control group 2), and GSH inhibitor (Control group 3), and compared with the control group (medium without any substances). Western blot analysis validated PBK expression levels (ELs) in various cell groups. Additionally, cell viability and proliferation were evaluated using methyl tetrazolium (MTT) assays and 5-Bromo-2'-deoxyuridine (BrdU) incorporation assays. The results revealed proton absorption peaks for HPAA at 2.78 ppm, 3.21 ppm, and 3.49 ppm, while RVG and HPAA-RVG exhibited characteristic absorption peaks at 23.653 min and 23.584 min, respectively, with peak areas of 4,856.6 and 6,927.3 for RVG. The nanoparticle sizes were 50-100 nm for HPAA-RVG and 100 nm for HPAA/RVG/PBK-siRNA, displaying spherical morphology and uniform size distribution. The average potential of HPAA-PEG was lower than that of HPAA (*P*<0.05), and HPAA-RVG showed dramatically lower potential than HPAA (*P*<0.001). At 8 hours, Daoy cells displayed higher endocytosis rates versus ONS-76 cells (*P*<0.05). The transfection rates of HPAA-RVG in both ONS-76 and Daoy cells were higher than those of HPAA, with Daoy cells showing higher transfection rates than HPAA (*P*<0.05). Under HPAA-RVG treatment, AChR levels in ONS-76 cells were significantly lower than those in Daoy cells (*P* < 0.05). Compared to the control group, the PBK protein expression levels, cell survival rates, and the number of cells in the proliferative phase were significantly reduced in Control group 1, the PEI group, and the HPAA/siRNA group in both ONS-76 and Daoy cells, with the ONS-76 cells in the HPAA/siRNA group showing the lowest values among these groups (*P* < 0.05). In summary, the findings indicated that the tumor microenvironment-responsive nanocomposite HPAA/RVG/PBK-siRNA selectively inhibited PBK expression in Daoy medulloblastoma cells, showcasing potential applicability in medulloblastoma therapy.

## Introduction

Medulloblastoma is a rare malignant intracranial tumor derived from the embryonic nervous system, primarily occurring in children and adolescents 1,2. Despite its low incidence, the treatment remains a major challenge due to the heterogeneity and diverse clinical presentations 3. Subgroups of medulloblastoma, including classic, desmoplastic/nodular, and large cell/anaplastic, exhibit significant pathological differences, leading to varied clinical presentations 4,5. Extensive research has highlighted the role of PDZ-binding-kinase (PBK), a PSD95/Discs-large/ZO-1 domain (PDZ)-binding kinase, as a crucial protein kinase in various cancers 6-8. The high expression level (EL) of PBK correlates closely with phenomena such as cell proliferation, metastasis, and drug resistance, making it an imperative therapeutic target in cancer 9. Nevertheless, conventional treatment methodologies face limitations in targeting PBK, including uneven systemic drug distribution and significant toxicity concerns 10.

Adoption of nanotechnology in the medical field has shown remarkable progress recently, offering new hope for cancer treatment. Nanomaterials have been extensively investigated and utilized in drug delivery systems, offering a precise, targeted, and efficient approach to cancer therapy 11. Research outcomes in the field of medulloblastoma have highlighted the significant potential of nanomedicine 12. With the continuous advancement of nanotechnology, targeted therapy for the tumor microenvironment becomes feasible. The tumor microenvironment encompasses cells, extracellular matrix, and various dissolved molecules surrounding tumor cells, playing a critical role in tumor development and treatment response 13,14. Through precise preparation and design, nanotechnology enables precise targeting of the tumor microenvironment, thereby enhancing the specificity and efficacy of treatment 15. Tumor microenvironment-responsive nanotherapeutic drugs represent a novel class of anti-cancer medications that can efficiently target specific molecules or cells within the tumor microenvironment, leading to highly effective tumor treatment 16. Leveraging the characteristics of the tumor microenvironment, these drugs achieve broad-spectrum, low toxicity, and reduced drug resistance by blocking interactions between tumor microenvironment and tumor cells, thereby inhibiting tumor cell growth 17. In this study, small interfering RNA (siRNA) targeting PBK was chosen as the therapeutic agent, and nanotechnology was utilized to enhance its stability and bioavailability *in vivo*, thereby achieving specific responsiveness to the tumor microenvironment.

With the deepening of molecular biology research, medulloblastoma has been classified into different molecular subgroups, including Sonic Hedgehog signaling pathway (SHH), WNT signaling pathway, Group 3, and Group 4 18. Among them, the SHH signaling pathway plays a crucial role in medulloblastoma, particularly in distinguishing different histological subgroups. The SHH subgroup is usually associated with a favorable prognosis, while non-SHH subgroup exhibit more invasive and poor prognostic features 19,20. For these different subgroups of medulloblastoma, recent studies have increasingly focused on using precise targeted treatment methods to improve therapeutic outcomes. Therefore, understanding the microenvironmental differences in different histological subgroups of medulloblastoma is crucial for the development of personalized nanotherapeutic strategies. However, comparative studies on the efficacy of targeted nanotherapeutics targeting PBK in the tumor microenvironment response across different histological subgroups of medulloblastoma are currently lacking.

**Figure 1:**
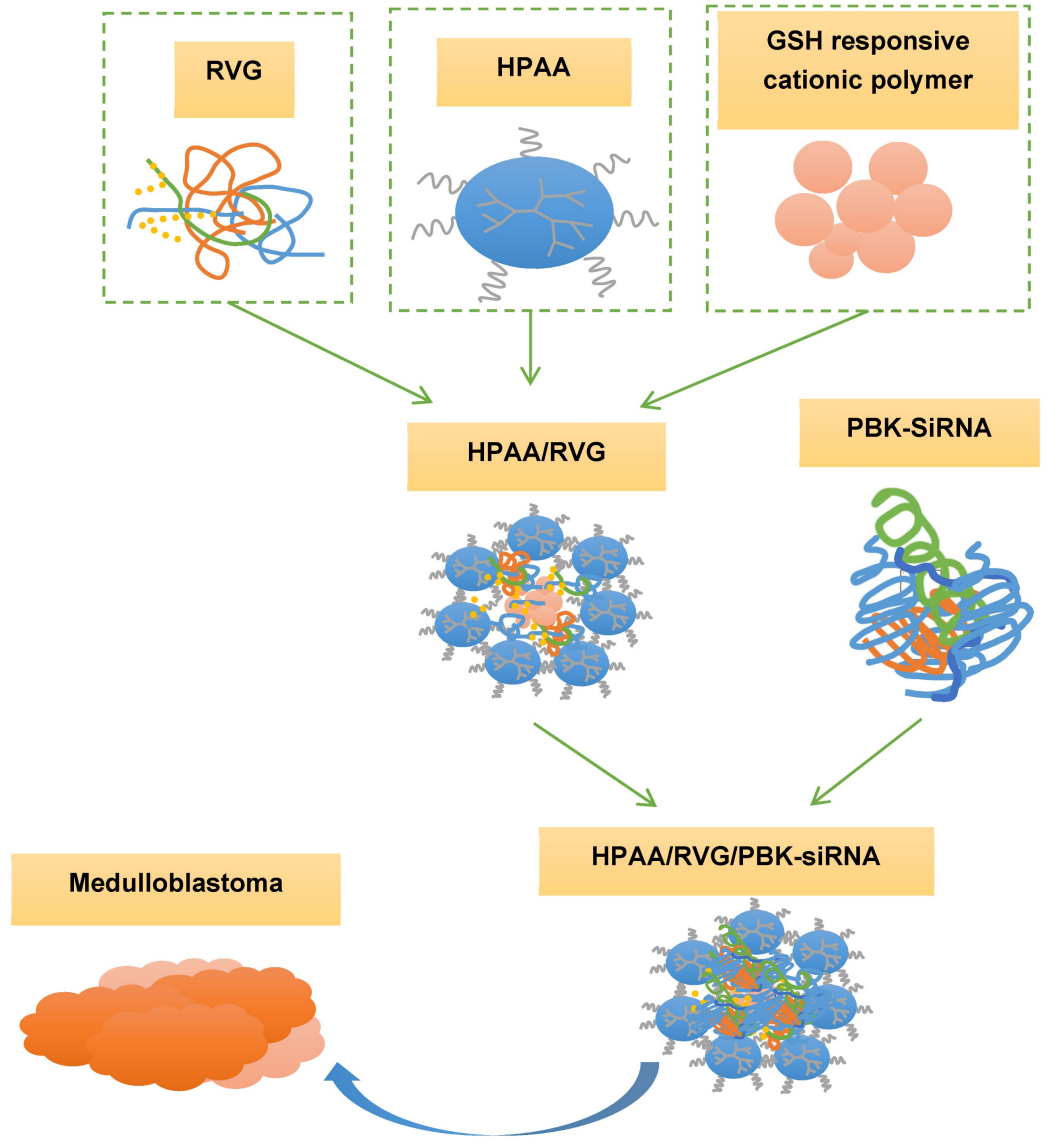
Synthesis and adoption of HPAA/RVG/PBK-siRNA complex.

The rabies virus glycoprotein (RVG) has been widely employed as a neuronal targeting ligand due to its specific binding to neurons rich in acetylcholine receptors, and it facilitates rapid entry of coupled compounds into nerve cells 21,22. Glutathione (GSH)-responsive cationic polymers demonstrate a notable advantage in gene delivery by swiftly releasing cargo under high GSH concentrations in the tumor microenvironment 23,24. Thus, in this study, RVG functioned as a specific targeting molecule, while GSH served as a microenvironmental stimulus within the hyperbranched polymer polyamide amine (HPAA). HPAA is loaded with PBK-siRNA, forming the HPAA/RVG/PBK-siRNA complex (Figure 1). The aim was to compare the therapeutic efficacy of tumor microenvironment-responsive nanotherapeutic drugs targeting PBK among various subgroups of medulloblastoma. This comparison aimed to provide crucial theoretical and practical foundations for the clinical adoption of tumor microenvironment-responsive nanotherapeutic drugs targeting PBK. It also offered new insights and strategies for personalized treatments for patients with various subgroups of medulloblastoma, ultimately contributing to enhancing the prognosis and survival rates of individuals with medulloblastoma.

## Materials and Methods

### HPAA synthesis

Ultrapurified water (Shanghai Hetai Instrument Co., Ltd., China) was utilized to dissolve 1.76 g of NaOH (Shanghai Boqu Instrument Co., Ltd., China). A mixture of 6.6 mL each of pre-cooled acryloyl chloride (Shanghai Aladdin Biochemical Technology Co., Ltd., China) and dichloromethane (Guangzhou Chemical Reagent, China) was prepared. The combination of these solutions was slowly applied to a solution of cystamine dihydrochloride (Shanghai Aladdin Biochemical Technology Co., Ltd., China) with a concentration of 0.225 g/mL using a syringe (Shandong Zhushi Pharmaceutical Group Co., Ltd., China). The mixture was stirred at 400 rpm under ice-bath conditions until the exothermic reaction subsided. After the removal of the ice bath, stirring continued at 800 rpm for 6 hours at 25 °C. Pre-cooled dichloromethane was poured into the mixture to completely dissolve the resulting white adhesive product. The solution was transferred into a separating funnel for extraction with sodium bicarbonate (Suzhou Dongbai Chemical Co., Ltd., China) and sodium chloride (Shandong Yongrui Salt Chemical Co., Ltd., China). The collected dichloromethane layer yielded a white powder after vacuum drying, named acryloyl-cystamine (CEA).

CEA, weighing 0.64 g, was mixed with a 3:1 volume ratio of methanol (Tianjin Concord Technology Co., Ltd., China) and water (containing 200 mmol/L CaCl_2_ (China National Pharmaceutical Group, China)) in a 10 mL solution. Stirring was carried out under N_2_ protection until complete dissolution. Ethylenediamine (Shanghai Aladdin Biochemical Technology Co., Ltd., China) was slowly applied (164 μL) and the reaction occurred at 50°C for 36 hours. Subsequently, 300 μL of piperazine was supplemented, and the reaction continued for an additional 12 hours. The pH was adjusted to 3-4 using HCl (Wuxi Haoteng Environmental Protection Technology Co., Ltd., China). After dialysis and freeze-drying, a light-yellow solid, identified as HPAA, was obtained.

### HPAA RVG and HPAA/RVG/PBK siRNA synthesis

A total of 200 mg of Maleimide (MAL)-polyethylene glycol (PEG)-N- n-succinyl ester (NHS) (MAL-PEG-NHS) (Shanghai Aladdin Biotech Company, China) was dissolved in phosphate buffered solution (PBS) with pH 7.4. HPAA was applied to it at a molar ratio of 1:10 (PEG: HPAA) and allowed to react at 25 °C in the absence of light for 2 hours. A 1000,0000 molecular weight cut-off ultrafiltration tube (Beijing Zeping Technology Co., Ltd., China) was used and centrifuged at 4 °C and 4,000 rpm/min using an Eppendorf centrifuge (USA) for 20 minutes. The centrifugation was repeated 1-2 times. The liquid in the centrifuge tube was freeze-dried to obtain a powder product, named HPAA-PEG.

HPAA-PEG, weighing 50 mg, was dissolved in a PBS solution at a pH range of 6.5-7.0. Sulfhydryl modified RVG (RVG-SH) (Shanghai Chutai Company, China), weighing 2 mg, was dissolved in a separate PBS solution. The RVG-SH solution was slowly dripped into the HPAA-PEG solution using a syringe within 30 minutes, and the mixture was stirred at 25 °C in the absence of light for 48 hours. The resulting reaction product was dialyzed using a membrane (Shanghai Yuanye Biotechnology Co., Ltd., China) with a 20,000 molecular weight cutoff and subsequently freeze-dried to obtain a white solid product, referred to as HPAA-RVG.

HPAA-RVG, weighing 2 mg, was dissolved in ultrapure water to prepare a solution of specific concentration, which was mixed with siRNA and PBK-siRNA (Suzhou Gene Pharma Company, China) at a mass ratio of 60:1, respectively. HPAA/RVG/siRNA and HPAA/RVG/PBK-siRNA complexes loaded with siRNA and PBK siRNA were obtained after incubation at 25 °C for 30 minutes.

### Characterization analysis

(1) Nuclear magnetic resonance (NMR) (Bruker-500 type, Brooke Company, Germany) spectroscopy analysis was implemented. 5 mg of CEA was dissolved in deuterated chloroform (CDCl3) (Shanghai Wentong Technology Co., Ltd., China). Additionally, 2 mg samples of HPAA, HPAA-PEG, and MAL-PEG-NHS were dissolved in 500 μL of deuterium oxide (D2O) (Shanghai Zhongwei Chemical Co., Ltd., China). The solutions were transferred to clean NMR tubes for NMR spectroscopy analysis.

(2) For high-performance liquid chromatography (HPLC) analysis of RVG and HPAA-RVG products, 2 mg each of RVG and HPAA-RVG were dissolved in 200 μL of acetonitrile (Hebei Yujinkun Technology Co., Ltd., China). C-18 columns (Hangzhou Weimipai Technology Co., Ltd., China) were utilized with acetonitrile and water as the mobile phase at a flow rate of 1 mL/min. Peak areas and retention times were determined at 218 nm.

(3) Potentiometric analysis was performed by dissolving 1 mg each of HPAA, HPAA-RVG, and HPAA-PEG in 1 mL of double-distilled water. The potentials were measured at 25°C using a Malvern laser particle size analyzer (Zetasizer Nano-ZS, Malvern Instruments, UK).

(4) Morphological observation was performed. 100 μL of HPAA/RVG/siRNA and HPAA/RVG/PBK-siRNA solutions were dropped onto copper grids for observation of morphology and particle size using transmission electron microscope (TEM) (JEM-2010HT type, Japan Electronics Co., Ltd., Japan).

### Cell culture and passage

For cell culture, Daoy and ONS-76 cell (ATCC, USA) lines were retrieved from frozen stocks using a pipette tip and transferred into 3 mL centrifuge tubes containing Dulbecco's modified eagle medium (DMEM) (Shanghai Yuanpei Biotechnology Co., Ltd., China) complete growth medium (containing 10% fetal bovine serum (FBS), 1% penicillin/streptomycin (P/S), and 1% non-essential amino acids (NEAA) (Gibco, USA)). Gentle pipetting was performed five times, followed by centrifugation at 25 °C for 5 minutes. The cell pellet was collected, resuspended in 1 mL DMEM, and then cultured at 37 °C in a CO_2_ incubator (Thermo Fisher 371 type, Thermo Fisher Scientific, USA). The old medium was removed after 48 hours, and cells were gently rinsed with sterile PBS buffer. Fresh medium was applied to maintain optimal conditions for cell growth. Subsequently, the medium was changed every three days to ensure a conducive environment for cell proliferation. Passaging was performed after seven days of culture.

Cell passaging was performed after seven days of cultivation, when cells reached the appropriate confluence. The culture medium was aspirated, and a mixture of 1 mL of 0.25% Trypsin (Gibco, USA) and 0.05% Ethylenediaminetetraacetic Acid (EDTA) (Gibco, USA) was added to facilitate cell separation. Once cells exhibited a rounded morphology, digestion was stopped, and the detached cell suspension was collected into a centrifuge tube. Suspension was distributed into new culture flasks at a ratio of 1:4. Fresh medium was applied to maintain cells under constant conditions of 5% CO_2_ at 37 °C.

### Endocytosis test

Using an endocytosis assay 25, the specific targeting efficiency of HPAA-RVG in Daoy and ONS-76 cells was quantitatively analyzed. The specific procedures were as follows: 20 mg of HPAA-RVG and 1 mg of Cyanine 5 (Cy-5) (Gibco, USA) were dissolved in PBS, gently stirred for 2 hours, the solution was collected, and dialyzed in a 500 molecular weight cutoff dialysis bag for 2-3 days until the solution became clear and transparent. The solution was filtered, freeze-dried, and reconstituted in sterile water (Thermo Fisher Scientific (China) Co., Ltd. (China Technology Company), China) for further use. Daoy and ONS-76 cells were seeded in a 24-well plate (Thermo Fisher Scientific (China) Co., Ltd. (China Technology Company), China) at 5×10^4^ cells/well, and incubated for 0.5 h, 1 h, 2 h, 4 h, and 8h. Subsequently, 1 mg/mL of HPAA-RVG was applied to the cells. 10 hours later, the culture medium was discarded and PBS was used for washing three times, and then cells were detached by suspending in an appropriate amount of PBS. Flow cytometry analysis was conducted using a flow cytometer (Beckman Coulter, Inc., USA), and data analysis was performed using FlowJo (Beijing Huanzhong Reagent (China) Technology Co., LTD., China). Approximately 10,000 average events per sample were collected during the flow cytometry analysis. The reliability and statistical significance of the results were ensured by using the average values from multiple repeated experiments.

### Cell transfection test

To detect the transfection efficiency of different concentrations of HPAA/RVG/PBK-siRNA in Daoy and ONS-76 cells, flow cytometry was used with Cy5 labeling. Cells were seeded at 5×10^4^ cells/well in a 24-well plate. Following 48 h incubation, the culture medium was replaced with optimized minimum essential medium (opti-mem) (Youbibio (Shanghai) Trading Co., Ltd., China) and starved for 2 hours. HPAA and HPAA-RVG were incubated separately with siRNA and PBK-siRNA at various mass ratios (10:1, 20:1, 40:1, 80:1, 120:1), while Methoxy-PEG polyethylene imine (PEI-25k) (Shanghai Aladdin Biochemical Technology Co., Ltd., China) served as the positive control, and a group without materials acted as the negative control. Following 2 h incubation, the medium was replaced, and the resulting HPAA/PBK-siRNA (HPAA), HPAA-RVG/PBK-siRNA (HPAA-RVG), PEI-25k/PBK-siRNA (PEI), and PBK-siRNA (Ctrl) were added to the 24-well plate containing Daoy and ONS-76 cells, followed by an additional 24-48 hours of incubation. Then, medium was aspirated, and the wells were rinsed thrice with pre-chilled PBS, cells were detached, suspended in PBS, and immediately analyzed using FCM with experimental data analyzed using *FlowJo* (three replicates per sample).

### Cell grouping and treatments

Daoy and ONS-76 cells were seeded in 6-well plates at a density of 2 × 10^5^ cells per well. After 24 hours of incubation, the old medium was removed, and the following treatments were applied:

Control group was treated with medium containing no substances.

HPAA/RVG group (HPAA/RVG carrier control) was treated with HPAA/RVG nanocarriers lacking PBK-siRNA to assess the carrier's effect on PBK.

shNTC group (shNTC control) was transfected with Dharmacon™ non-targeting siRNA (Thermo Fisher Scientific, USA).

Control group 1 (PBK-siRNA Control) was treated with medium containing PBK-siRNA.

Control group 2 (AChR inhibitor control) was treated with medium containing AChR inhibitor.

Control group 3 (GSH inhibitor control was treated with medium containing GSH inhibitor.

HPAA/siRNA group was treated with complete medium containing HPAA/RVG/PBK-siRNA (HPAA/siRNA).

PEI group (positive control) was treated with PEI-25k/PBK-siRNA (PEI) complexes.

### Western blot (WB)

The effectiveness of different PBK-siRNAs was validated using Western blotting to identify the optimal gene suppression. Cells from each group were seeded into 6-well plates and incubated in a cell culture incubator for 48 hours. Afterward, the plate was washed twice with PBS, and then protein extraction reagent (Thermo Fisher Scientific (China) Co., Ltd. (China Technology Company), China) was applied. The plate was incubated on ice for 5 minutes, and cells and extraction solution were collected. Following centrifugation at 4°C and 160,000 rpm/min for 20 minutes, the supernatant collected post-centrifugation was utilized for protein blot analysis. Protein concentration was assessed using the Bicinchoninic acid (BCA) Protein Quantitative Kit (Nanjing Keygen Company, China), followed by protein separation via sodium dodecyl sulfate-polyacrylamide gel electrophoresis (SDS-PAGE) (Shanghai Beyotime Company, China). After electrophoresis, proteins were transferred onto a polyvinylidene fluoride (PVDF) (Shenzhen Datanghong Technology Co., Ltd., China) membrane, which was blocked using a 5% skimmed milk (Hebei Jiuxing Chemical Products Co., Ltd., China) solution at 25 °C for 10 minutes. After the membrane was washed with Tris-buffered saline containing Tween-20 (TBST) (Saint-Bio, China), the membrane was incubated overnight at 4°C with monoclonal anti-PBK antibody (catalog number AB1234, dilution 1:1000), anti-AChR antibody (catalog number CD2325, dilution 1:1500), and polyclonal anti-GAPDH antibody (catalog number CD5678, dilution 1:2000) (Morey Biosciences, USA). Following TBST washing, the membrane was then incubated for 2 hours at 25°C with horseradish peroxidase-conjugated secondary antibody (dilution 1:5000, catalog number EF9012, Beijing Bersee Technology Co., Ltd., China). Subsequently, the membrane was rinsed with TBST and underwent chemiluminescent detection using an enhanced chemiluminescence (ECL) detection kit (Beijing Biolab Technology Co., Ltd., China) following the manufacturer's instructions. The gel image was captured using a gel documentation system (Shanghai Jinpeng Analytical Instrument Co., Ltd., China), and the relative grayscale values of the target proteins were quantified using* ImageJ* (National Institutes of Health, USA). Each group of samples underwent at least three replicates of experiments.

### Cell survival rate detection

Daoy and ONS-76 cells were evenly seeded in a 6-well culture plate to promote attachment and growth. Specifically, each well was seeded with 1×10^5^ Daoy cells and 5×10^4^ ONS-76 cells. Following the drug treatment, 3-(4,5-dimethyl-2-thiazolyl)-2,5-diphenyl-2-H-tetrazolium bromide (MTT) (Shanghai Shangbao Biotechnology Co., Ltd., China) solution was applied, and cells were cultured for 24 hours to form purple formazan crystals. Then, the culture medium was removed and a dimethyl sulfoxide (DMSO) solvent (Shanghai Aladdin Biochemical Technology Co., Ltd., China) was added to completely dissolve the purple crystals. Absorbance was then measured using a microplate reader (550 type, bio-rad company, USA) at a wavelength of 570 nm to reflect cell viability. Repetition of experiments and calculation of averages were performed to minimize experimental errors. At least three independent replicates were performed for each group of samples.

### Cell proliferation detection

The 5-Bromo-2'-deoxyuridine (BrdU) (Beijing Biolab Technology Co., Ltd., China) labeling method was employed 26 to analyze the impact of HPAA/RVG/PBK-siRNA and PEI-25k/PBK-siRNA complexes, along with PBK-siRNA, on the proliferation of Daoy and ONS-7 cells. Cell culturing and drug treatment were consistent with Section 2.8. Following the drug treatment, BrdU solution was introduced, allowing it to substitute for thymidine in the newly synthesized DNA strands. Subsequently, cells were fixed, subjected to DNA denaturation, and stained using specific anti-BrdU antibodies. Finally, staining results were visualized under a microscope (ZEISS Carl Zeiss (Shanghai) Management Co., Ltd., China), and the proportion of BrdU-positive cells was calculated, reflecting the number of cells in the proliferative phase.

### Statistical analysis

The quantitative experimental results were presented in the format of mean ± standard deviation. One-way analysis of variance using *SPSS 22.0* was conducted for comparing all the data to assess statistical significance. Differences were statistically significant when *P*<0.05.

## Results

### NMR analysis of CEA, HPAA, HPAA-PEG, MAL-PEG-NHS, HPAA-RVG

**Figure 2:**
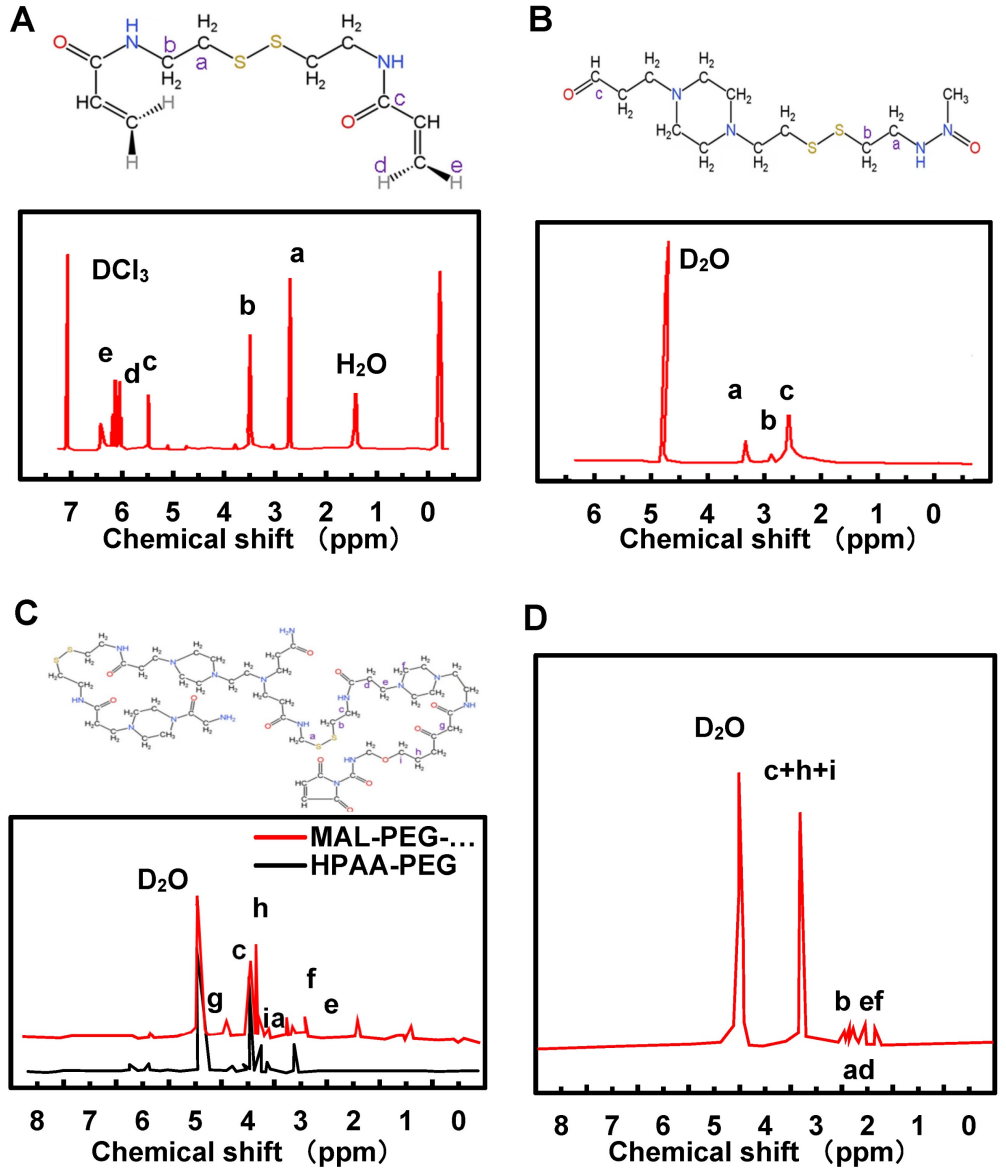
NMR spectrums of CEA (A), HPAA (B), HPAA-PEG (C), MAL-PEG-NHS (C), and HPAA-RVG (D).

NMR was employed for the detection and analysis of HPAA, HPAA-PEG, MAL-PEG-NHS, and HPAA-RVG. The results are depicted in Figure 2A~D. In Figure 2A, the chemical shifts of CEA were observed with a varying number of hydrogen atoms (H), labeled as a (4H), b (4H), c (2H), d (2H), and e (2H). The NMR spectra exhibited corresponding peak values δ at 2.9 ppm, 3.68 ppm, 6.22 ppm, 5.68 ppm, and 6.34 ppm, respectively. Figure 2B illustrates the proton absorption peaks a, b, c at different positions in HPAA with peak values δ of 2.78 ppm, 3.21 ppm, and 3.49 ppm, respectively. The NMR characterization analysis of HPAA-PEG and MAL-PEG-NHS is presented in Figure 2C. The proton absorption peaks of HPAA were observed between 2.51-3.52 ppm, while in HPAA-PEG and MAL-PEG-NHS, the characteristic absorption peak δ of PEG methylene (-CH_2_) was found at 3.62 ppm, and the characteristic absorption peak δ of maleimide double bond in MAL was observed at 5.98 ppm. Additionally, the characteristic absorption peak δ of maleimide double bond in MAL-PEG-NHS was slightly shifted to the right compared to HPAA-PEG. Figure 2D indicates that unlike HPAA-PEG, HPAA-RVG does not exhibit characteristic absorption peaks of maleimide double bond, while the absorption peaks corresponding to other functional groups remain present.

### Analysis of HPLC results for RVG and HPAA-RVG

**Figure 3:**
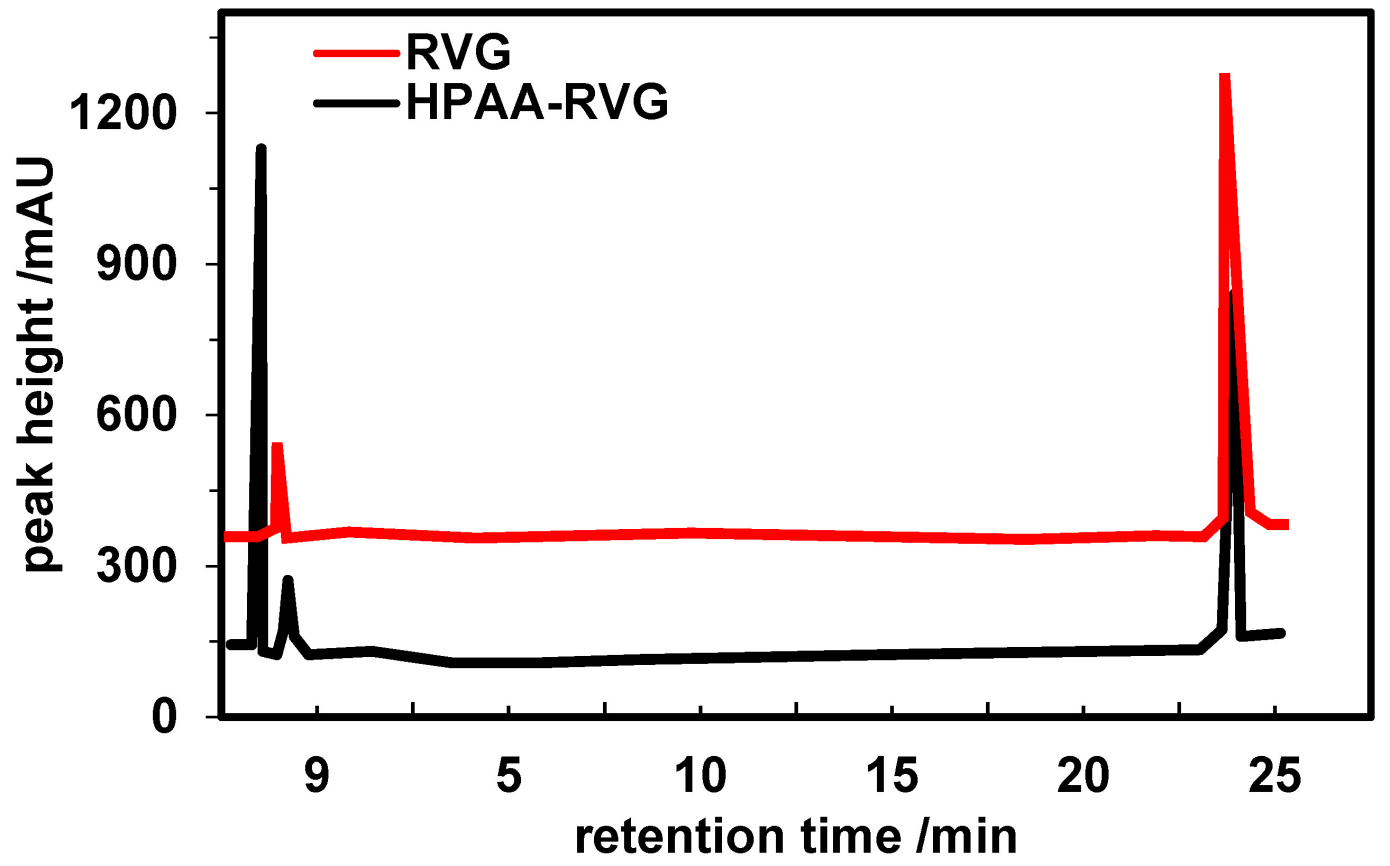
HPLC spectra of RVG and HPAA-RVG.

Further validation of the characteristics of RVG and HPAA-RVG was conducted using the HPLC method. The results are depicted in Figure 3. Peaks characteristic of RVG were observed at 23.653 minutes and 23.584 minutes for RVG and HPAA-RVG, respectively. The peak areas corresponding to the RVG characteristic peaks for RVG and HPAA-RVG were measured as 4,856.6 and 6,927.3, respectively.

### Morphological observation and zeta potential analysis of HPAA, HPAA-RVG, and HPAA/RVG/PBK-siRNA

**Figure 4:**
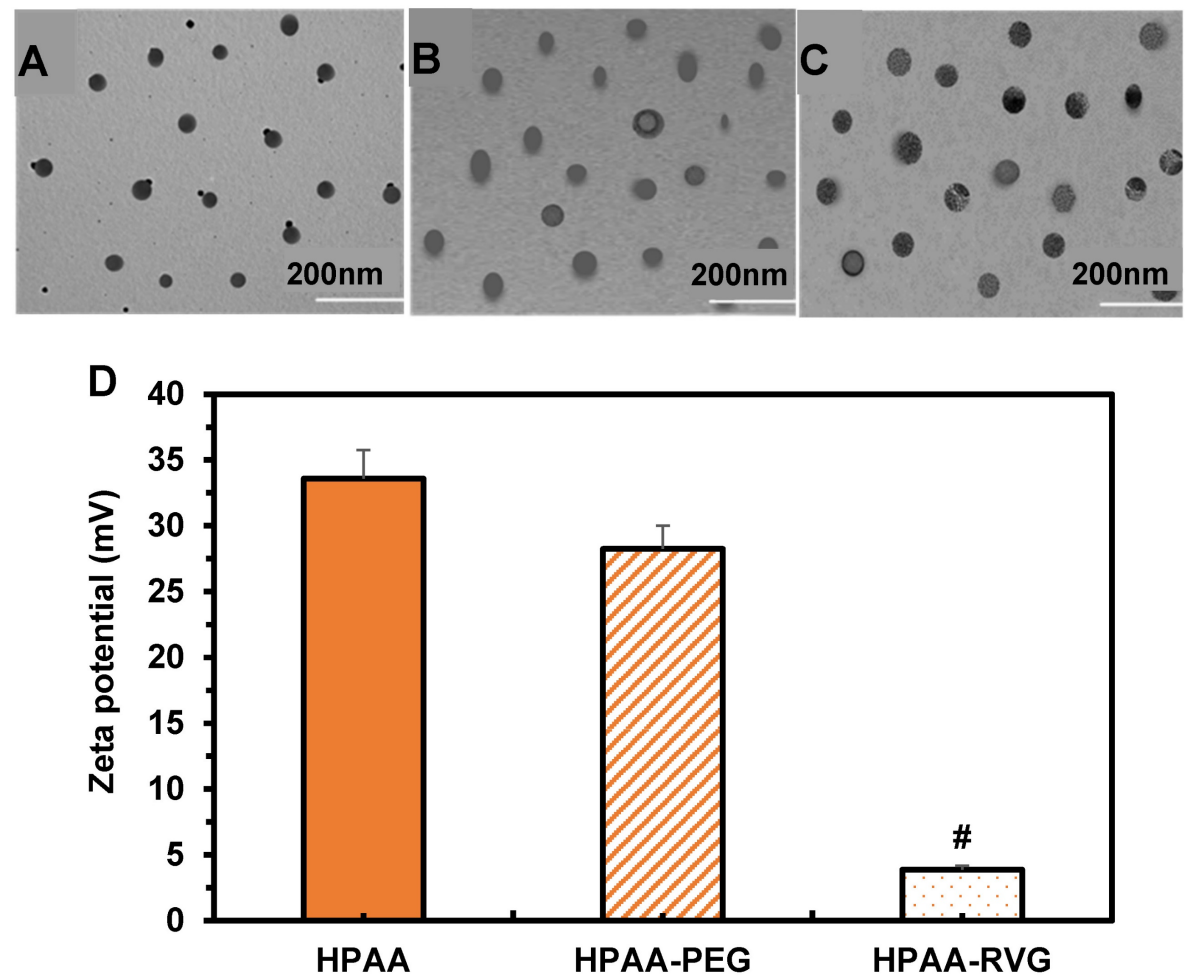
** TEM morphology and zeta potential comparison of HPAA (A), HPAA-RVG (B), and HPAA/RVG/PBK-siRNA (C) and Zeta potential comparison (D).** #*P*<0.001 vs. HPAA.

TEM was employed to visualize the morphology and particle size of HPAA, HPAA-RVG, and HPAA/RVG/PBK-siRNA. The results in Figure 4A~C revealed that HPAA exhibited a size range of approximately 20-80 nm, displaying a well-defined spherical structure with compact morphology. HPAA-RVG nanoparticles exhibited a size range of approximately 50-100 nm, maintaining a uniform spherical shape with even particle distribution. Upon gene complexation, HPAA/RVG/PBK-siRNA nanoparticles displayed a size of approximately 100 nm, maintaining a compact nanospherical morphology. The study utilized a laser nanometer particle size analyzer to measure the zeta potentials of HPAA, HPAA-PEG, and HPAA-RVG (Figure 4D). The average zeta potentials of HPAA, HPAA-PEG, and HPAA-RVG were 33.58 ± 2.18 mV, 28.25 ± 1.75 mV, and 3.87 ± 0.29 mV, respectively. The average zeta potential of HPAA-PEG was lower than that of HPAA (*P* < 0.05), while HPAA-RVG showed a significantly lower zeta potential compared to HPAA (*P* < 0.001).

### Endocytosis analysis of HPAA/RVG nanoparticles in cells

**Figure 5:**
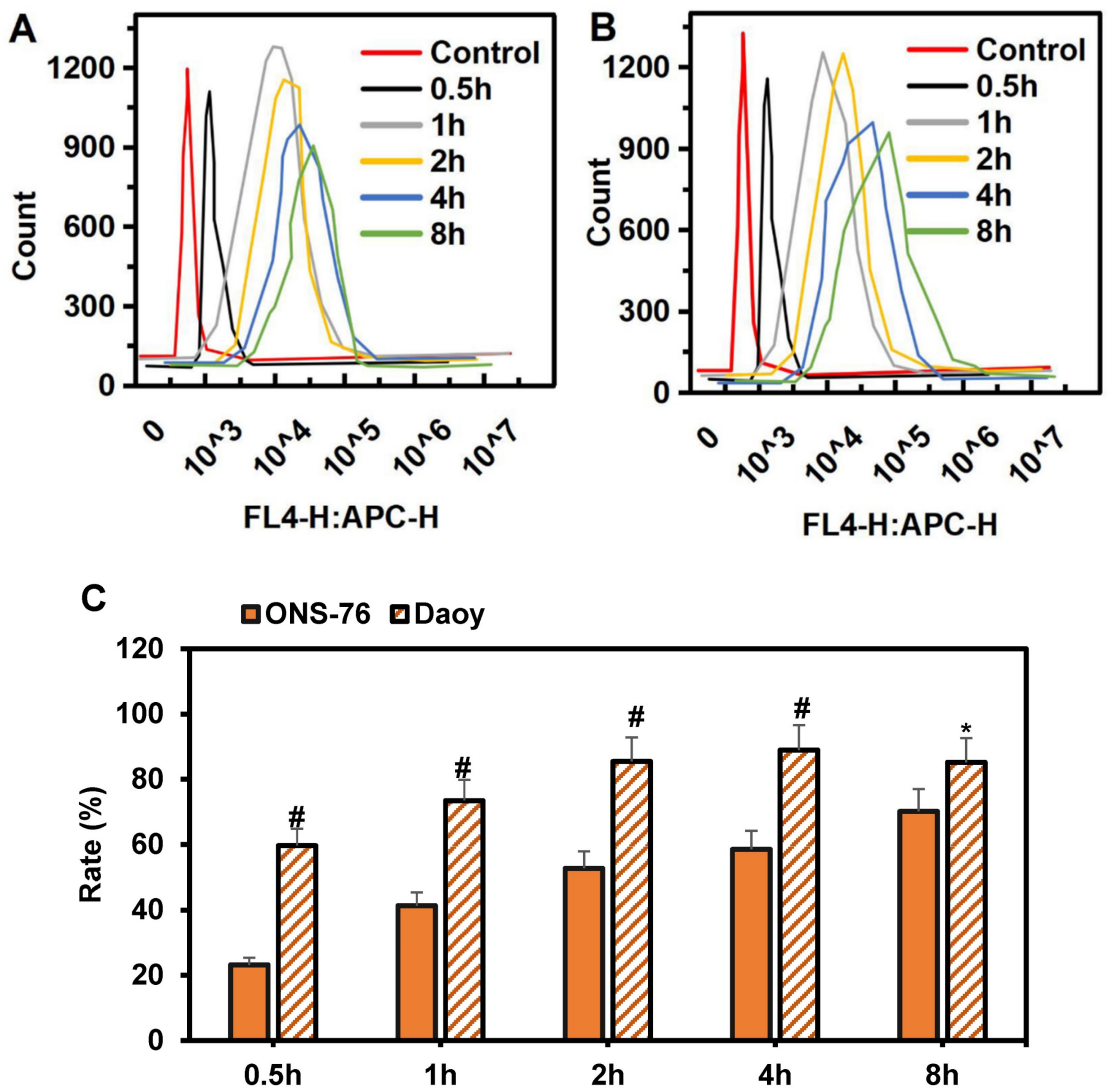
** Comparison of intracellular endocytosis of Cy-5-labeled HPAA/RVG nanoparticles at various time periods in ONS-76 cells and Daoy cells.** (A) ONS-76 cells; (B) Daoy cells; (C) Endocytosis rate. *P<0.05 vs. ONS-76 cells; #*P*<0.01 vs. ONS-76 cells.

The specific targeting effect of HPAA-RVG nanoparticles on medulloblastoma was analyzed using Daoy and ONS-76 cells, as demonstrated in Figure 5A~B. At 0.5 hours, minimal internalization of HPAA-RVG nanoparticles was observed within both Daoy and ONS-76 cells, resulting in relatively weak fluorescence intensity. However, with prolonged incubation, the quantity of internalized HPAA-RVG nanoparticles increased, accompanied by enhanced fluorescence intensity, demonstrating a clear time-dependent trend. Subsequently, the endocytosis rates of HPAA-RVG nanoparticles within Daoy and ONS-76 cells at different incubation times were further statistically analyzed, as presented in Figure 5C. At 0.5 hours, the internalization rates of Daoy and ONS-76 cells were 59.62±5.16 and 23.15±2.26, respectively. Prior to 4 hours, the internalization rate of Daoy cells was significantly higher than that of ONS-76 cells (*P*<0.01). By 8 hours, the endocytosis rates of Daoy and ONS-76 cells were 85.19±7.37 and 70.14±6.84, respectively, with Daoy cells exhibiting a higher endocytosis rate compared to ONS-76 cells (*P*<0.05).

### Cell transfection analysis

**Figure 6:**
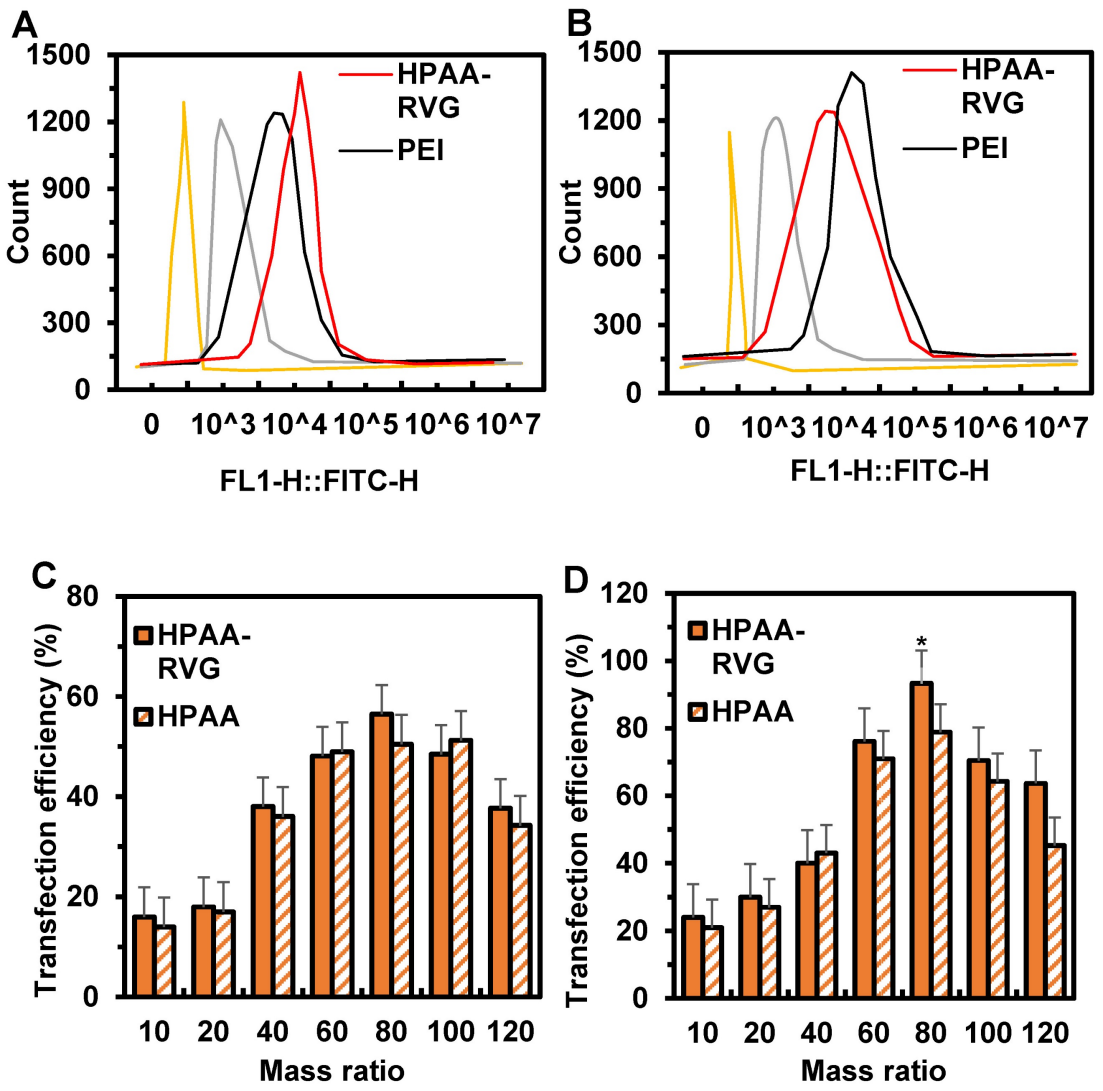
** Cell transfection in Ctrl group, PEI group, HPAA group, and HPAA-RVG group and in HPAA group and HPAA-RVG group under different mass ratios of PBK-siRNA and si NA-FAM**. (A) Number of transfected ONS-76 cells; (B) Number of transfected Daoy cells; (C) Transfection rate of ONS-76 cells; (D) Transfection rate of Daoy cells. *P<0.05 vs. HPAA.

The transfection efficiency of HPAA/RVG in ONS-76 and Daoy cells is depicted in Figure 6A~B. The fluorescence intensity of HPAA/RVG was highest in both ONS-76 and Daoy cells, while the fluorescence intensity of PEI and HPAA was significantly lower than that of HPAA-RVG. The peak fluorescence intensity of HPAA/RVG in Daoy cells was higher than that in ONS-76 cells. The cellular transfection efficiency of HPAA and HPAA-RVG with PBK-siRNA at different mass ratios is illustrated in Figure 6C~D. When the mass ratios of HPAA and HPAA-RVG to PBK-siRNA were 10, 20, and 40, the transfection rates of HPAA-RVG in ONS-76 and Daoy cells were lower than those of HPAA. There was no statistically significant difference in transfection rates between HPAA-RVG and HPAA in both ONS-76 and Daoy cells (*P*>0.05). When the mass ratio of HPAA and HPAA-RVG to PBK-siRNA was 80, the transfection rates of HPAA-RVG and HPAA reached their maximum values in ONS-76 and Daoy cells, respectively, at 56.46±7.96 and 50.45±6.58, 93.29±8.92 and 78.84±6.89. The transfection rate of HPAA-RVG in both ONS-76 and Daoy cells was higher than that of HPAA, with the transfection rate of HPAA-RVG being higher than that of HPAA in Daoy cells (*P*<0.05). However, when the mass ratio of HPAA and HPAA-RVG to PBK-siRNA was greater than 80, the transfection rates of HPAA and HPAA-RVG gradually decreased.

**Figure 7:**
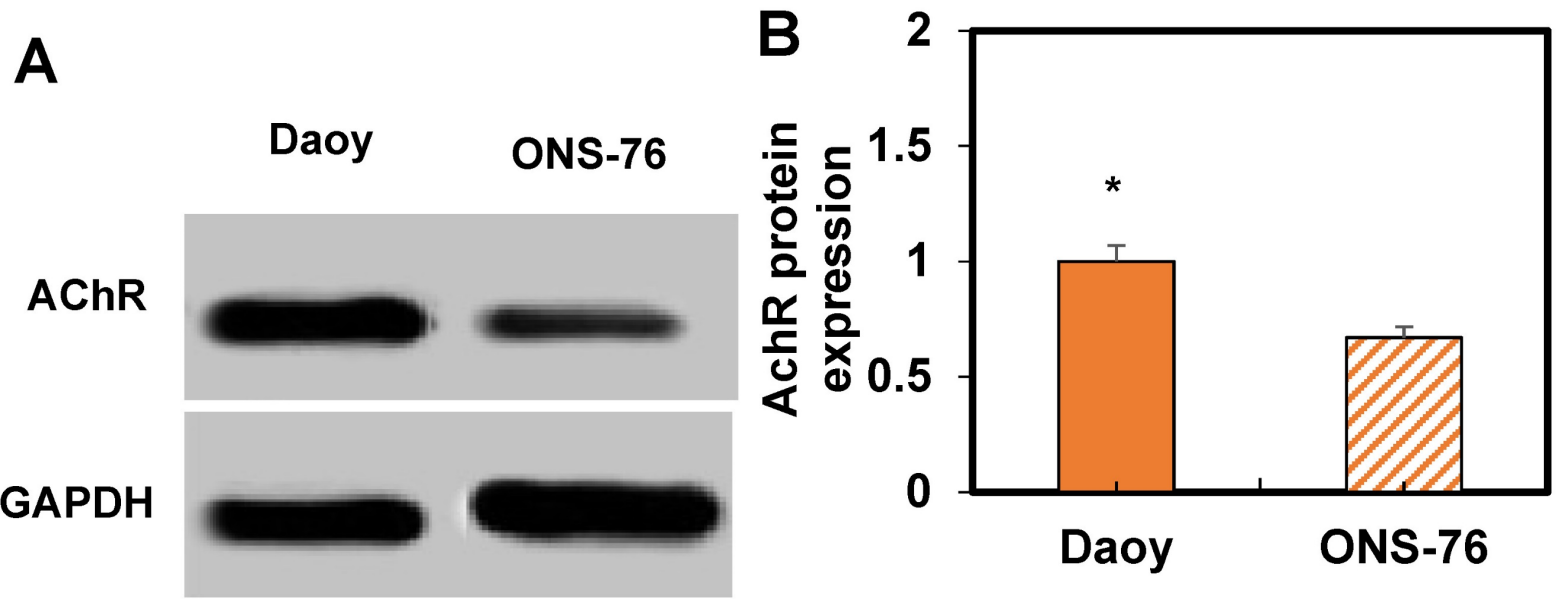
** WB results of AChR expression.** A: WB image; B: Comparation of expression levels. *P<0.05 vs. ONS-76 cells.

Based on these results, WB analysis was performed to assess AChR expression in ONS-76 and Daoy cells treated with HPAA/RVG (Figure 7). It can be observed that Daoy cells exhibit higher AChR expression compared to ONS-76 cells (*P* < 0.05).

### Western blotting results for PBK protein expression levels in cells from each group

**Figure 8:**
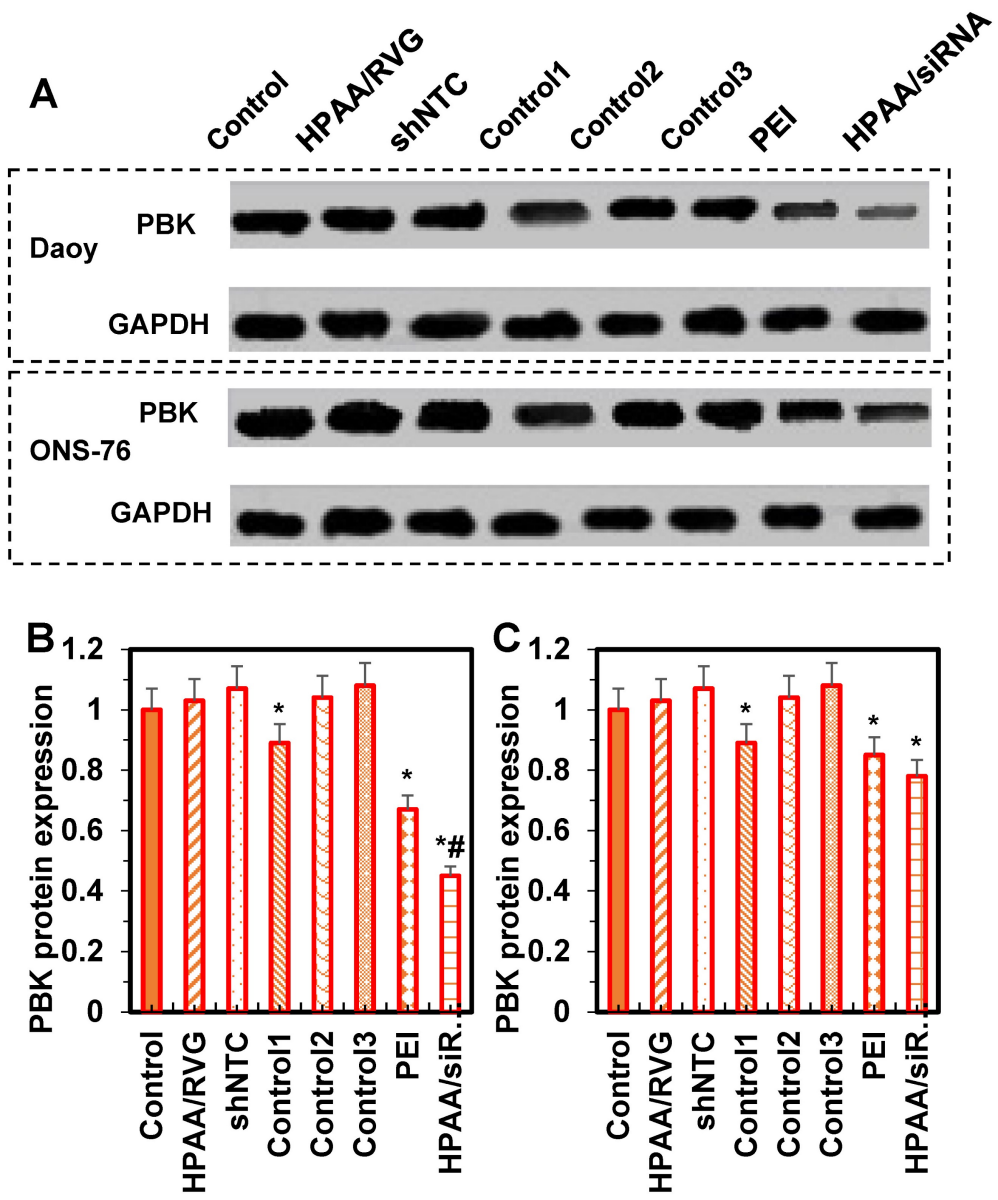
** Comparison of protein ELs among various groups.** (A) WB imprint; (B) Daoy cells; (C) ONS-76 cells. *P < 0.05 vs. the control group; #*P* < 0.05 vs. the PEI group and Control group 1.

The statistical analysis of PBK protein expression levels in ONS-76 and Daoy cells is shown in Figure 8. Compared to the control group, there were no significant differences in PBK protein expression levels among the HPAA/RVG group, shNTC group, Control group 2, and Control group 3 (*P* > 0.05). In contrast, PBK protein levels were significantly lower in Control group 1, the PEI group, and the HPAA/siRNA group, with the HPAA/siRNA group showing the lowest levels in ONS-76 cells compared to Control group 1 and the PEI group (*P* < 0.05). These results indicate that the HPAA/RVG carrier does not significantly affect PBK protein levels, while PBK-siRNA exhibits specific inhibitory effects on PBK protein expression, suggesting minimal interference for subsequent studies.

### Analysis of the effects of HPAA/PBK siRNA, PEI-25k/PBK siRNA, and PBK siRNA on cell survival and proliferation

Based on the above results, further analyses were conducted on the cell viability and proliferation of Daoy and ONS-76 cells in the Control group, Control group 1, Control group 2, Control group 3, PEI group, and HPAA/siRNA group.

Regarding cell viability, compared to the control group, there were no significant differences in cell viability of Daoy cells between Control group 1 and Control group 2 (*P* > 0.05). However, the viability was significantly reduced in Control group 1, the PEI group, and the HPAA/siRNA group, with the HPAA/siRNA group exhibiting the lowest viability (*P* < 0.05).

**Figure 9:**
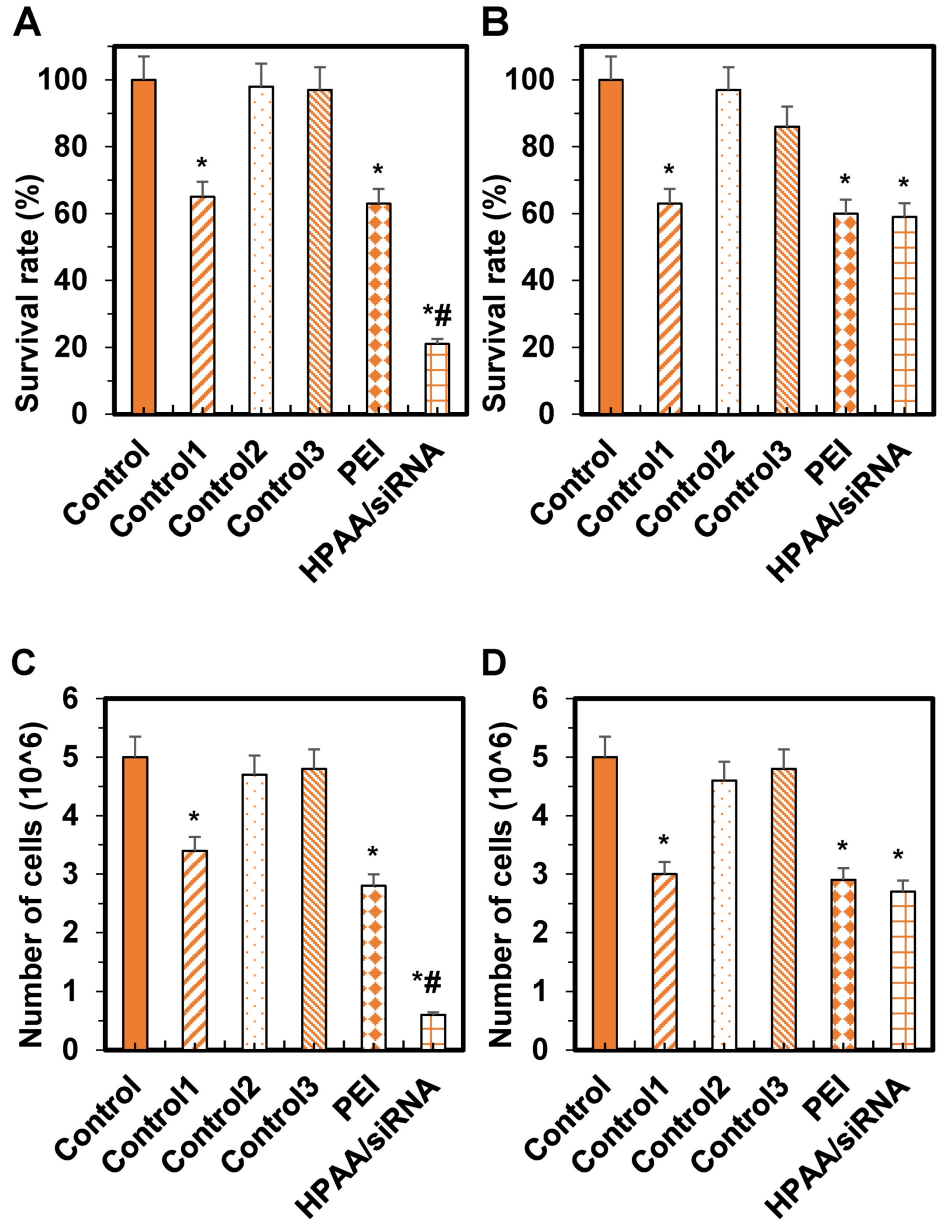
** Comparison of cell survival rates and the number of proliferating cells in various groups.** (A) Daoy cell survival rate; (B) ONS-76 cell survival rate; (C) Daoy cell number in the proliferative phase; (D) ONS-76 cell number in the proliferative phase. *P < 0.05 vs. the control group; #*P* < 0.05 vs. the PEI group and Control group 1.

For ONS-76 cells, compared to the control group, there were no significant differences in cell viability between Control group 1 and Control group 2 (*P* > 0.05). Nonetheless, viability was significantly decreased in Control group 1, the PEI group, and the HPAA/siRNA group (*P* < 0.05), with no significant differences among Control group 1, the PEI group, and the HPAA/siRNA group (*P* > 0.05) (Figures 9A~B).

Regarding cell proliferation, compared to the control group, there were no significant differences in the number of Daoy cells in the proliferative phase between Control group 1 and Control group 2 (*P* > 0.05). The number of proliferating cells was reduced in Control group 1, the PEI group, and the HPAA/siRNA group, with the HPAA/siRNA group showing the lowest number of proliferating cells (*P* < 0.05).

For ONS-76 cells, compared to the control group, there were no significant differences in the number of cells in the proliferative phase between Control group 1 and Control group 2 (*P* > 0.05). The number of proliferating cells was reduced in Control group 1, the PEI group, and the HPAA/siRNA group (*P* < 0.05), but there were no significant differences among Control group 1, the PEI group, and the HPAA/siRNA group (*P* > 0.05) (Figures 9C~D).

## Discussion

Given the heterogeneity and clinical diversity of medulloblastoma, significant challenges arise in its clinical management. Recently, PBK has attracted considerable attention for its potential in treating various medulloblastoma subgroups. In this study, nanotechnology was utilized to develop targeted PBK-responsive nanotherapeutic agents tailored to the tumor microenvironment. These agents were applied across different subgroups of medulloblastoma to assess their therapeutic efficacy.

Firstly, the preparation of HPAA, HPAA-PEG, MAL-PEG-NHS, and HPAA-RVG was evaluated. The successful synthesis of CEA was confirmed by NMR through chemical shifts and peak identification. Within HPAA molecules, distinct chemical environments and various types of protons were observed. In comparison to HPAA-PEG, NMR characterization revealed a rightward shift in the characteristic absorption peak of MAL-PEG-NHS. This shift may be attributed to the influence of functional group migration on HPAA 27. Upon reaction between the maleimide double bond of MAL and PEG-succinimide ester in MAL-PEG-NHS, alterations in chemical environments and interatomic interactions occurred, leading to changes in chemical shifts 28. These results confirm the successful synthesis of HPAA-PEG. In contrast to HPAA-PEG, HPAA-RVG lacks characteristic absorption peaks of maleimide double bonds, possibly due to the addition reaction of maleimide double bonds in HPAA-PEG with RVG bearing a spacer group to form HPAA-RVG 29. The absence of maleimide double bonds on RVG confirms the successful addition of maleimide double bonds to RVG, thereby validating the successful synthesis of HPAA-RVG. HPLC analysis revealed that the retention time and position of the characteristic absorption peak of RVG in both RVG and HPAA-RVG were essentially identical, indicating the presence of RVG within the polymer. However, differences were observed in the peak areas of the RVG characteristic absorption peaks between RVG and HPAA-RVG, possibly due to variations in the content or concentration of RVG in RVG and HPAA-RVG. Combining the results from NMR and HPLC, it was concluded that HPAA-RVG was successfully synthesized. TEM analysis confirmed the successful complexation of PBK-siRNA with HPAA-RVG as the gene carrier, as evidenced by observations of morphology and particle size. Furthermore, the study revealed that HPAA/RVG/PBK-siRNA nanoparticles targeted to PBK had minimal impact on the morphology and particle size distribution of HPAA material itself. Additionally, the morphology and particle size distribution of HPAA-RVG nanoparticles complied with the requirements for gene delivery 30. One of the prerequisites for HPAA-RVG as a gene carrier is its ability to electrostatically adsorb and bind with negatively charged genes 31. Laser particle size analysis revealed that the average zeta potential of HPAA-PEG was lower than that of HPAA (*P*<0.05), and the average zeta potential of HPAA-RVG was significantly lower than that of HPAA (*P*<0.001). This confirms a notable decrease in the average zeta potential of HPAA, HPAA-PEG, and HPAA-RVG, attributed to the negatively charged RVG peptide segment 32, which, upon grafting onto HPAA, shields the negative charge on its surface. This research verified the positive charge of HPAA-RVG, enabling it to adsorb genes with negative charges. Simultaneously, the introduction of PEG and RVG targeting peptides renders the surface potential of HPAA more neutral, which holds crucial significance for subsequent gene delivery endeavors.

The acetylcholine receptor (AChR) is a membrane protein that plays a crucial role in transmitting nerve impulses and initiating muscle contraction at neuromuscular junctions 33. In certain cases, certain types of cancer cells on the cell surface may express or secrete AChR. Medulloblastoma is a neuroembryonic tumor originating from neuroectodermal cells 34. Early-stage medulloblastoma cells typically exhibit characteristics resembling those of neuronal precursor cells, including the expression of neurites and neuron-specific markers 35. Current research suggests that highly differentiated mature medulloblastoma cells express AChR, and these cells expressing AChR can autonomously grow and proliferate via the ACh signaling pathway 36. RVG, as a neuron-targeting ligand, can specifically bind to acetylcholine receptors 37. Building upon these studies, this research evaluates the impact of HPAA-RVG nanoparticles on Daoy and ONS-76 cells.

The internalization experiments demonstrated a notable time-dependent increase in the quantity of HPAA-RVG nanoparticles internalized by cells with prolonged internalization time. Notably, prior to 4 hours, the internalization rates of Daoy cells were significantly higher than those of ONS-76 cells (*P*<0.01), and at 8 hours, the internalization rate of Daoy cells remained higher than that of ONS-76 cells (*P*<0.05). These findings indicated that during the initial stages, Daoy cells exhibited a higher capacity for engulfing HPAA-RVG nanoparticles. As time progressed, Daoy cells sustainably demonstrated higher engulfment capacity, while ONS-76 cells exhibited relatively lower engulfment capacity. At 8 hours, both Daoy and ONS-76 cells showed an increasing trend in internalization rates; however, Daoy cells still exhibited a higher engulfment capacity. This discrepancy may be attributed to differences in the ELs of AChR and cellular characteristics between the two cell types. Daoy cells, typical of medulloblastoma, often exhibit elevated levels of AChR expression, resulting in a substantial presence of AChR on their cell membrane. In contrast, ONS-76 cells may possess lower levels of AChR expression. RVG, functioning as a neuron-targeting ligand, can selectively bind to AChR. Given the higher levels of AChR expression in Daoy cells, the targeting efficacy of HPAA-RVG nanoparticles during internalization may be more pronounced. Conversely, ONS-76 cells, potentially due to lower ELs of acetylcholine receptors, may exhibit comparatively weaker targeting effects. Under HPAA-RVG treatment, the AChR levels in ONS-76 cells were found to be lower than in Daoy cells (*P* < 0.05), consistent with the results of the study. Nonetheless, ONS-76 cells still demonstrate internalization effects, likely attributed to the small particle size of HPAA-RVG nanoparticles themselves. Regardless of whether targeting ligands are grafted, these nanoparticles can be internalized via endocytosis due to their diminutive size.

Cell transfection analysis revealed that the fluorescence intensity of HPAA/RVG was highest in ONS-76 and Daoy cells, whereas the fluorescence intensity of PEI and HPAA was significantly lower than that of HPAA-RVG. This may be attributed to the lack of inherent cell-targeting properties in PEI and HPAA, hindering their efficient cellular uptake. The diminished fluorescence intensity may reflect their relatively lower rates of engulfment and transfection efficiency during the cellular internalization process. Additionally, the peak fluorescence intensity of HPAA/RVG in Daoy cells surpassed that in ONS-76 cells, possibly attributable to inherent cellular characteristics. This further underscores the targeting effect of RVG, enhancing the internalization efficiency of HPAA and consequently augmenting gene transfection efficiency. Further analysis of the cell transfection efficiency of HPAA and HPAA-RVG with PBK-siRNA at different mass ratios revealed that when the mass ratio of HPAA and HPAA-RVG to PBK-siRNA was 80, the transfection rates of HPAA-RVG were higher than those of HPAA in both ONS-76 and Daoy cells, with the transfection rate of HPAA-RVG in Daoy cells being higher than that of HPAA (*P*<0.05). However, when the mass ratio exceeded 80, the transfection rates of both HPAA and HPAA-RVG gradually decreased. This suggests that HPAA-RVG/PBK-siRNA achieves optimal gene transfection efficacy at a mass ratio of 80:1.

The PBK protein is an essential protein in the human body, playing a crucial role in cell proliferation and DNA replication processes 38. *In vivo*, PBK gene expression primarily occurs in proliferative tissues and organs, such as embryonic and tumor tissues. This indicates that PBK may play a significant regulatory role in cell proliferation. During DNA replication, PBK binds to the PCNA protein, forming a complex. PCNA serves as an auxiliary factor for DNA polymerases δ and ε, facilitating DNA synthesis. Upon binding to PCNA, PBK can modulate the process of DNA replication and participate in the regulation of DNA synthesis. Besides its involvement in cell proliferation and DNA replication, the PBK protein is also associated with processes such as cell cycle regulation, cell division, and apoptosis. Studies have shown that high expression of the PBK protein in tumor cells is associated with tumor progression and prognosis 39. Therefore, PBK protein may serve as a potential target for tumor therapy and diagnosis. This study found that PBK protein expression levels were significantly reduced in ONS-76 and Daoy cells in Control group 1, the PEI group, and the HPAA/siRNA group, with PBK expression being lower in ONS-76 cells in the HPAA/siRNA group compared to Control group 1 and the PEI group (*P* < 0.05). This indicates that both PEI and HPAA/siRNA inhibit PBK protein expression. PBK protein is associated with cell proliferation and cell cycle regulation, and its reduction may inhibit proliferation in Daoy cells. The HPAA carrier demonstrated higher efficacy in suppressing PBK protein expression. For ONS-76 cells, both PEI and HPAA carriers effectively inhibited PBK protein expression and cell proliferation, though this inhibition was less pronounced compared to Daoy cells. Additionally, in Daoy cells, the HPAA/siRNA group had the lowest cell viability and number of proliferative cells, indicating the strongest inhibitory effect. For ONS-76 cells, the HPAA/siRNA group also showed significantly lower viability and proliferative cell numbers compared to the control group, with no significant differences between the PEI group and Control group 1. Combined with the PBK Western blotting results, the study confirms the close association between high PBK expression and cancer cell survival and proliferation, thereby enhancing the accuracy of the findings.

## Conclusions

This work aimed to investigate the therapeutic efficacy of a PBK-targeted tumor microenvironment-responsive nanotherapeutic agent in medulloblastoma Daoy and ONS-76 cells. The preparation and characterization of the tumor microenvironment-responsive nanocomplex HPAA/RVG/PBK-siRNA were conducted, followed by analysis of its transfection and EL in Daoy and ONS-76 cells using FCM and WB assays. The results indicated a marked inhibition of PBK protein EL in Daoy cells by this nanocomplex, potentially exerting inhibitory effects on cell proliferation. Nevertheless, the study was limited by the lack of validation of the therapeutic efficacy of this nanocomplex in animal models, requiring further research and verification. In conclusion, the tumor microenvironment-responsive nanocomplex HPAA/RVG/PBK-siRNA showed potential for treating medulloblastoma, providing a theoretical and practical basis for the clinical adoption of PBK-targeted tumor microenvironment-responsive nanotherapeutics.

## Abbreviations

MB: Medulloblastoma
GSH: Glutathione
PBK: PDZ-binding-kinase
PSD95: Postsynaptic density protein 95
RNA: Ribonucleic acid
siRNA: Small interfering RNA
HPAA: Hyperbranched polyamide amine
RVG: Rabies virus glycoprotein
PEG: Polyethylene glycol
NHS: N-hydroxysuccinimide
MAL: Maleimide
TEM: Transmission electron microscope
NMR: Nuclear magnetic resonance
HPLC: High-performance liquid chromatography
PBS: Phosphate buffered saline
DMEM: Dulbecco's modified eagle medium
FBS: Fetal bovine serum
P/S: Penicillin/streptomycin
NEAA: Non-essential amino acids
Cy5: Cyanine 5
FCM: Flow cytometry
MTT: Methyl thiazolyl tetrazolium
DMSO: Dimethyl sulfoxide
SDS-PAGE: Sodium dodecyl sulfate-polyacrylamide gel electrophoresis
PVDF: Polyvinylidene fluoride
TBST: Tris-buffered saline with Tween 20
ECL: Enhanced chemiluminescence
WB: Western blot
BrdU: 5-Bromo-2'-deoxyuridine
AChR: Acetylcholine receptor
